# The Role of Intestinal Mucosal Barrier in Autoimmune Disease: A Potential Target

**DOI:** 10.3389/fimmu.2022.871713

**Published:** 2022-07-01

**Authors:** Jia An, Yuqing Liu, Yiqi Wang, Ru Fan, Xiaorong Hu, Fen Zhang, Jinhua Yang, Junwei Chen

**Affiliations:** ^1^ Department of Rheumatology, The Second Hospital of Shanxi Medical University, Taiyuan, China; ^2^ Department of Microbiology and Immunology, Shanxi Medical University, Taiyuan, China; ^3^ Department of Internal Medicine, Central Hospital of Xinghualing District, Taiyuan, China

**Keywords:** intestinal mucosal barrier, zonulin pathway, intestinal microbiome, toll-like receptors signal pathway, autoimmune diseases

## Abstract

Autoimmune diseases are a series of diseases involving multiple tissues and organs, characterized by the over production of abnormal multiple antibodies. Although most studies support that the impaired immune balance participates in the development of autoimmune diseases, the specific pathogenesis of it is not fully understood. Intestinal immunity, especially the intestinal mucosal barrier has become a research hotspot, which is considered to be an upstream mechanism leading to the impaired immune balance. As an important defense barrier, the intestinal mucosal barrier regulates and maintains the homeostasis of internal environment. Once the intestinal barrier function is impaired under the effect of multiple factors, it will destroy the immune homeostasis, trigger inflammatory response, and participate in the development of autoimmune diseases in the final. However, the mechanism of the intestinal mucosal barrier how to regulate the homeostasis and inflammation is not clear. Some studies suggest that it maintains the balance of immune homeostasis through the zonulin pathway, intestinal microbiome, and Toll-like receptor signaling pathway. Our review focused on the composition and the function of the intestinal mucosal barrier to describe the research progress of it in regulating the immune homeostasis and inflammation, and also pointed that the intestinal mucosal barrier was the potential targets in the treatment of autoimmune diseases.

## Introduction

Autoimmune diseases, such as rheumatoid arthritis (RA), systemic lupus erythematosus (SLE), type 1 diabetes (T1D), and inflammatory bowel disease (IBD) are a group of the chronic and life-threatening inflammatory diseases, characterized by the imbalance of immune homeostasis and the production of abnormal autoantibodies. The etiology of autoimmune diseases is complex, including genetic, hormonal, and environmental factors (1), but the exact mechanism is unknown. Numerous studies support that the breakdown of immune tolerance is one of the key mechanisms to promote the pathogenesis and progression of autoimmune diseases (2), but the specific mechanism of impaired immune tolerance is still unclear. The number of patients with autoimmune diseases has been increasing year by year, therefore it is urgent to explore the mechanism of the breakdown of immune tolerance and find the potential targets for the treatment of autoimmune diseases. The challenges in the diagnosis and treatment of autoimmune diseases are to identify the early events that cause autoimmunity, which inspires researchers to further study the pathogenesis of autoimmune diseases. In recent years, the intestinal immunity has been a hot research topic (3, 4). With diving deep into it, increasing evidences suggest that the intestinal mucosal barrier, as an important component of intestinal immunity, is not only a medium for the absorption and exchange of substances between the organism and the environment but also prevents external antigens from entering into the body (5). The complete composition and function of the intestinal mucosal barrier function are essential for maintaining the immune homeostasis. Once the intestinal mucosal barrier is impaired, external antigens will invade into the body through the damaged intestinal mucosal barrier to induce and aggravate systemic inflammatory response, which leads to the destroyed autoimmune tolerance and imbalance of immune homeostasis to exacerbate the progression of autoimmune diseases such as RA, SLE, and IBD (6–8). Thus, the normal intestinal mucosal barrier may be the upstream factor in maintaining immune homeostasis, and the function of intestinal mucosal barrier is associated with the zonulin pathway, intestinal microbiome, and Toll-like receptors (TLRs) signal pathway, which are the important triggers for the intestinal mucosal barrier to regulate the immune system (9–11). If there is any problem in the zonulin pathway, intestinal microbiome, and TLRs signal pathway, it will affect the function of the normal intestinal mucosal barrier. However, the specific regulatory mechanism remains to be further studied. In this review, we demonstrated the composition and function of the intestinal mucosal barrier and described the regulatory mechanisms to discuss the role of the intestinal mucosal barrier function in the occurrence and development of autoimmune diseases, aiming to propose new directions on the pathogenesis and precise treatment of autoimmune diseases.

## Composition and Function of the Intestinal Mucosal Barrier

The intestinal mucosal barrier is the main defense line to against potentially harmful substances and infectious agents. It is composed of physical barrier, chemical barrier, immune barrier, and microbial barrier.

### Intestinal Physical Barrier

The intestinal physical barrier is an important part of the intestinal mucosal barrier, which is composed of mucus, intestinal epithelial cells, and tight junctions between intestinal epithelial cells (5, 12). And the intestinal epithelial cells, mainly including goblet cells, Paneth cells, and M cells, are the mainstay of the intestinal physical barrier and play a central role in host enteric defense (13). These cells not only have the capability of rapid proliferation and regeneration but also can secrete mucus, antimicrobial proteins (AMPs), and secreted immunoglobulin A (SIgA), which are the structural basis for maintaining intestinal chemical barrier, immune barrier, and other barrier function (14). Especially, mucus, a highly glycated mucin secreted by goblet cells, is an important component of the intestinal physical barrier. It covers the surface of intestinal epithelial cells and plays a physical isolation role to prevent the intestinal microbiome from directly acting on intestinal mucosa (15, 16). The experimental evidence showed that animals lacking mucus were susceptible to intestinal inflammation, which proved that mucus exerted the protective effect to prevent the entry and spread of pathogenic microbes (17). The continuity of the intestinal physical barrier depends on the presence of tight junctions between intestinal epithelial cells. Tight junctions consist of transmembrane proteins including claudin, occludin, zonula occludens 1 (ZO1) and cingulin (18), which maintain the structural integrity and biological functions of intestinal epithelial cells. These transmembrane proteins can regulate the intercellular space between intestinal epithelial cells to prevent bacteria, antigens and other substances in the intestinal cavity from entering the intestinal lamina propria, while promote the transport of nutrients (19). It has been confirmed that in pathological states, the dysfunction of the intestinal mucosal barrier caused by disordered tight junctions can lead to a large number of antigen molecules or microorganisms to further aggravate the inflammatory response through paracellular pathways (20). Therefore, the integrity of tight junctions is essential for avoiding activating immune cells and abnormal immune responses, which plays a key role in the stability of the internal environment.

### Intestinal Chemical Barrier

The intestinal chemical barrier is composed of gastric acid, bile, various digestive enzymes, lysozyme, and AMPs, which interact with each other to form a complex network (21). Gastric acid, secretions of gastric, pancreatic, and bile can degrade some microorganisms and antigens in a non-specific manner, while digestive enzymes and lysozymes have toxic effects on microorganisms by destroying their cell walls to exert bactericidal and bacteriolytic effects. AMPs are produced by intestinal epithelial cells, including α- defensins, β-defensins, RegIIIγ, and C-type lectins. And AMPs have broad-spectrum and high-efficiency bactericidal activity against bacteria, which can remove most of the microorganisms and prevent the access of antigens to host cells in the intestinal lumen to maintain the chemical barrier function (22, 23). The intestinal chemical barrier is a good supplement to the intestinal mechanical barrier, and plays an important role in maintaining intestinal micro ecological balance.

### Intestinal Immune Barrier

As an integral part of the gut tissue, the intestinal immune barrier is necessary to maintain homeostasis, which includes the innate immune system and the adaptive immune system (6, 24, 25).

The innate immune system is composed of antibacterial substances such as mucins and AMPs, which have the non-specific ability to eliminate harmful microorganisms and have been proved to play an important role in maintaining intestinal mucosal barrier function (6, 25). Especially, AMPs secreted by intestinal epithelial cells are important effector molecules in the innate immune system, which not only contribute to the local containment of pathogens but also exert the effect of anti-inflammatory and recruiting immune cells (26–28).

The adaptive immune system refers to gut-associated lymphoid tissue (GALT) and SIgA, which is one of the important components of the immune system (29). GALT mainly consists of multi-follicular lymphoid tissues, such as Peyer’s lymph nodes riched in lymph follicles, mesenteric lymph nodes (MLNs), isolated lymphoid follicles (ILF), and scattered lymphocytes in the lamina propria and epithelium of the mucosa (30–32). As the largest lymphoid tissue in the body, GALT recognizes foreign antigens and abnormal antigens in time by ingesting, processing, and presenting antigens. And after specifically recognizing foreign antigens, GALT promotes the production of cytokines and antibodies to coordinate with the immune response. Besides, it also activates T and B lymphocytes to establish effective adaptive immune response and induce the mucosal immune response or immune tolerance (31, 32). Another component of the adaptive immune system in the intestinal immune barrier is SIgA, which is the most abundant immunoglobulin in the body released by B cells (33). SIgA resides primarily on intestinal mucosal surfaces. It can interact with commensal bacteria by coating bacteria to form antigen-antibody complexes to neutralize the toxins produced by bacteria. And it also can stimulate the secretion of intestinal mucus to prevent bacteria from adhering to the intestinal mucosa and protect the intestines from the destruction of foreign antigens to avoid activating abnormal immune responses (34). In addition, SIgA also regulates the composition of the intestinal microbiome to help maintain the balance of the environment in the body, which is important for maintaining the normal function of the intestinal mucosal barrier (10).

The intestinal mucosal immune barrier coordinates with the immune response. It can eliminate the invaded pathogens through local immunity to avoid the excessive activation of the systemic immune response. When the intestinal immune barrier is damaged or abnormal, it will lost the ability to eliminate pathogens and destroy the intestinal homeostasis, which will lead to the unrestricted activation of the immune response, and cause the occurrence of various autoimmune diseases.

### Intestinal Microbial Barrier

There are a large number of microbiomes in the human gut, which are mainly composed of *Bacteroides*, *Firmicutes*, *Proteobacteria*, and *Actinomycetes*. The intestinal microbiome can regulate the digestion and the absorption of nutrients, and provide energy for intestinal epithelial cells (35). And at the same time, to avoid the harmful immune response caused by the intestinal microbiome, the host and the intestinal microbiome cooperate to evolve and adapt to each other through two-way communication, and gradually establish a mutually beneficial relationship (10, 36, 37). As a result, the interaction between the intestinal microbiome and the intestinal mucosa constitutes a micro-ecosystem, namely the intestinal microbial barrier. In normal conditions, the abundance and diversity of the intestinal microbiome are in a dynamic equilibrium, which is essential for maintaining and regulating the function of the intestinal mucosa barrier. The commensal microbiome is important for shaping the intestinal mucosal, and the complex interactions of the two can act on immune mediators to regulate the immune system (38). There is increasing evidence that the intestinal microbiome plays a key role in regulating and supporting the intestinal mucosa barrier (10, 39). On the one hand, the intestinal microbiomes prevent the invasion of pathogens and improve the resistance to the colonization of harmful or pathogenic bacterial species by releasing antibacterial substances. On the other hand, the intestinal microbiomes limits the over response of the intestinal immune system to antigens to maintain immune tolerance and homeostasis (10, 24). Therefore, the intestinal microbial barrier is an important protective barrier against pathogens. In addition, there are other pathogens in the gut such as enteric viruses, which also play a role in regulating the intestinal microbiome and the immune system. In the process of interaction with enteric viruses, intestinal epithelial cells gradually establish and improve the immune defense mechanisms to strengthen the function of the intestinal mucosa barrier well (40, 41).

**Figure 1:**
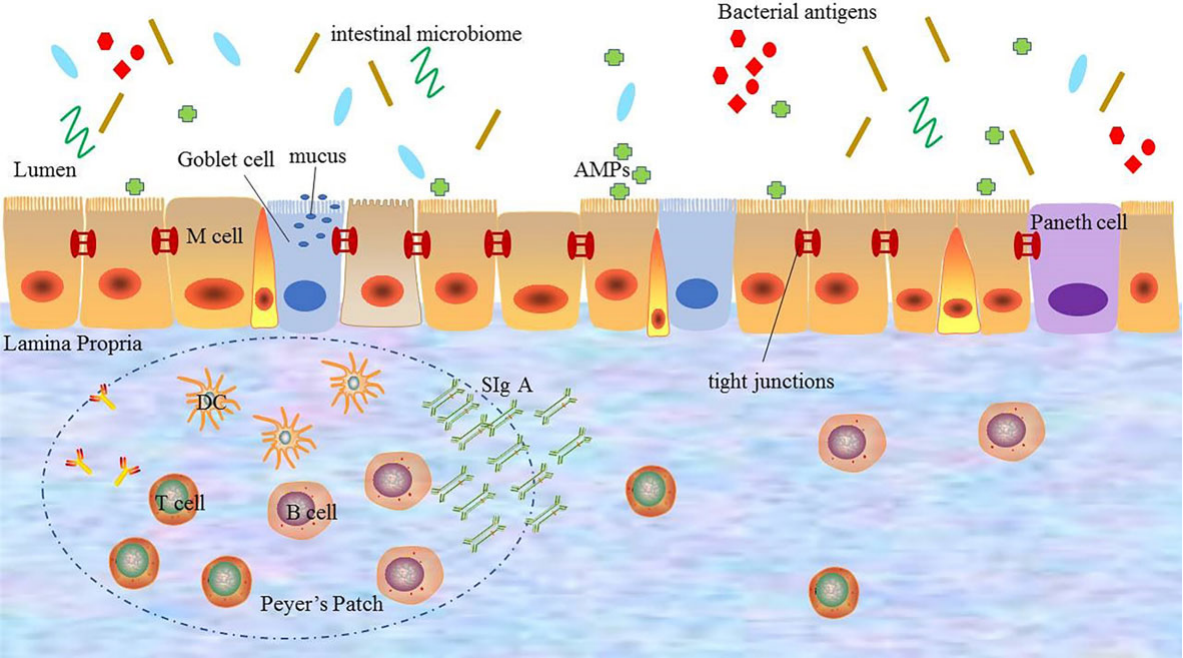
The composition of the intestinal mucosal barrier. The intestinal mucosal barrier includes physical barriers, chemical barriers, immune barriers, and microbial barriers. Each barrier interacts with each other to perform barrier functions. DC: dendritic cell; AMPs: antimicrobial proteins; SIgA: secreted immunoglobulin A.

In short, the normal intestinal mucosal barrier function is an important protective mechanism and defense system of the body. And only with the integrity and coordination of the each barrier, the intestinal mucosal barrier can fully play the role of defending against pathogenic microorganisms from invading, regulating the introduction of antigens and inhibiting inflammation (**Figure 1**).

## The Mechanism of the Intestinal Mucosal Barrier Regulating Body Homeostasis and Inflammation

As an important protective mechanism and defense system of the body, the intestinal mucosal barrier forms a strict regulatory interface between the immune system and the outside world to control the mucosal immune response and determine the balance between tolerance and immune response to a large extent. The intestinal mucosal barrier has a strict regulatory mechanism, which is the basis for ensuring the integrity and synergy of the intestinal mucosal barrier. Various factors, such as drug, infections and diet, not only destroy the function of intestinal mucosal barrier, but also affect the regulatory mechanism of it, resulting in the increase of intestinal permeability (6, 42), which cause intestinal bacteria, toxins, inflammatory mediators, and antigen products in the intestinal lumen to cross the intestinal wall into the circulation or other tissues, namely “translocation”. Translocation will activate a variety of inflammatory and immune cells abnormally to produce a large number of pro-inflammatory factors to cause the abnormal host immune response and further trigger the loss of immune tolerance to self-antigens (6, 7), which is related to the development of autoimmune diseases. At the same time, uncontrolled inflammatory responses in turn promote the destruction of the intestinal mucosal barrier and aggravate the disorders of immune and internal environmental homeostasis, which is a vicious cycle. In this process, the regulatory mechanism of the intestinal mucosal barrier is particularly important. However, the mechanism leading to the dysfunction of the intestinal mucosal barrier is not fully understood. Current studies indicate that the zonulin pathway, intestinal microbiome and TLRs signaling pathway can serve as the regulatory mechanisms of the intestinal mucosal barrier and participate in the regulation of homeostasis and inflammation in the body (9–11).

### Zonulin Pathway

Tight junction is a key structure to regulate intercellular molecular transport, and its integrity is one of the important factors to ensure the function of the intestinal mucosal barrier. Although steady progress has been made in the composition and function of tight junctions, the complex regulatory mechanisms are still incompletely clear. Zonula occludens toxin (Zot) is an enterotoxin elaborated by Vibrio cholera, which can open the intercellular tight junctions and lead to the breakdown of the intestinal mucosal barrier. With the study of Zot, an intestinal Zot homolog was discovered (43), and it was confirmed to be the same molecule as an inactive precursor of pre-haptoglobin 2(HP2) (44). The protein is zonulin, which is the only known physiological endogenous modulator of the tight junction (45).

Zonulin is a kind of 47 kDa paracrine protein secreted by intestinal epithelial cells and released into the lumen of the small intestine. It can interact with the surface receptor to modulate the opening of intestinal tight junctions quickly and reversibly by the zonulin pathway. The zonulin binds to the zonulin receptor of intestinal epithelial cells to activate the zonulin pathway. When the zonulin pathway is activated, it will transcribe and activate the epidermal growth factor receptor (EGFR) by the protease-activated receptor 2 (PAR2) activation peptides to further trigger protease-activated receptor leading to the separation of ZO-1 and occludin from the tight junction complex to promote the opening of intestinal mucosal barrier (44, 46). The secretion of zonulin depends on the adaptor protein myeloid differentiation primary response protein 88 (MYD88). In physiologic states, zonulin makes tight junctions open to promote the secretion of fluid in the intestinal lumen to effectively prevent bacterial colonization. But the opening of the tight junctions is transient and tightly modulated, once the zonulin signaling is over, the tight junctions resume a steady state again. However, in the pathological states, the pathogens or lipopolysaccharide (LPS) (the major cell wall component of gram-negative bacteria) (47) can act on the chemokine (C-X-C motif) receptor 3(CXCR3)-MYD88 signal pathway continuously, which leads to the over production of zonulin. And the excessive serum level of zonulin activates the zonulin pathway continuously to prolong the opening of the tight junctions and increase the permeability of the intestinal mucosal barrier, which causes the antigen trafficking through the intestinal mucosal barrier to activate immune cells to produce pro-inflammatory cytokines, ultimately leading to uncontrolled immune responses and loss of tolerance in individuals (9, 48). On the contrary, the use of zonulin antagonists can block the intestinal mucosal barrier dysfunction mediated by the zonulin pathway, which further suggests that the zonulin pathway plays an important role in regulating the intestinal mucosal barrier. Therefore, zonulin is considered to be a marker related to impaired intestinal mucosal barrier function.

The discovery of zonulin and the zonulin pathway has advanced the understanding of the complex mechanisms of tight junctions to regulate intestinal permeability, which may be the triggering event for autoimmune diseases. Some studies have demonstrated that zonulin is associated with the immune-inflammatory response. The study by Ciccia et al. (49) found that the level of zonulin in the serum of patients with ankylosing spondylitis (AS) was up-regulated, which influenced the expression of tight junctions between intestinal epithelial cells and ultimately induced the systemic immune response of AS. Coincidentally, the study by Tajik et al. (50)found that compared with healthy controls, patients with RA exhibited the increased intestinal permeability and the significantly increased level of zonulin, which induced the T cell-mediated mucosal inflammation to trigger arthritis by reducing the expression of tight junction proteins in the intestine. A study used a zonulin transgenic mouse (Ztm) model(characterized by increased small intestinal permeability)found that the Ztm model showed an altered gene expression profile of tight junctions compared to wild-type mice, which were compatible with the loss of intestinal mucosal barrier function (51). These studies all indicate that zonulin can regulate the intestinal mucosal barrier.

In brief, the breakdown of the intestinal mucosal barrier caused by the excessive signal transduction of the zonulin pathway or the high level of zonulin can participate in the immune response and lead to the abnormal activation of autoimmunity. Therefore, as important pathways for regulating tight junctions, zonulin and zonulin pathways are one of the important mechanisms for maintaining intestinal mucosal barrier function, which can be used as therapeutic targets to improve the disease activity of autoimmune diseases.

### The Intestinal Microbiome

As a potential regulator of autoimmune response, the intestinal microbiome has gradually become a frontier hot spot in immunology (52, 53), especially, the symbiosis and the interaction with organisms. The intestinal microbiome can interact with the intestinal mucosal barrier. On the one hand, the intestinal mucosal barrier provides the environment for the survival of the intestinal microbiome. On the other hand, the intestinal microbiome plays an important role in regulating the development and maturation of the intestinal mucosal barrier (10).

A study has found that the mucin secreted by the intestinal epithelial cells and the thickness of the mucus layer covering the mucosal surface in the germ-free mice was significantly reduced compared with conventionally housed mice. However, after the products of the intestinal microbiome in conventionally housed mice were administered to germ-free mice, the secretion of mucin and the thickness of the mucus layer were restored to the level of normal mice (54). It suggested that the secretion of mucus in the intestinal mucosal barrier depends on the intestinal microbiome. And the intestinal microbiome is closely related to the expression of tight junction proteins. In which the increase of *Oscillibacter* was negatively correlated with the mRNA expression of tight junctions protein ZO-1 (55), which showed that the intestinal microbiome was important to maintain the function and integrity of the intestinal mucosal barrier. In addition, the intestinal microbiome is also very important in maintaining the intestinal immune barrier. Study has confirmed that taking probiotics or increasing probiotics such as *Lactobacillus* and *Bifidobacterium* could restore the characteristics of RegIIIγ and control the overgrowth of bacteria (6). And compared with conventionally housed mice, the production and maturation of GALT in germ-free mice are affected, and the number of Peyer’s lymph nodes in the lamina propria is reduced, which all lead to the abnormal local immune tolerance (24). At the same time, as an important component of the intestinal microbiome, some enteric viruses also play a role in the development of the immune system by promoting the differentiation of T cells and up-regulating the anti-bacterial activities (56).

Therefore, the intestinal microbiome promotes the development and maturation of the intestinal mucosal barrier. The interaction between the intestinal microbiome and the intestinal mucosal barrier is essential to maintain the stability of the mucosal environment. The dynamic balance of the intestinal microbiome regulates the development and function of the human immune system through a variety of mechanisms.

### TLRs Signal Pathway

TLRs are a kind of cell transmembrane signal transduction protein in the innate immune system, which recognize the microbial-related molecular patterns (MAMPs) to trigger different immune responses (11). As the pattern recognition receptors (PRRs), the signal transduction pathways and immune regulation mechanisms of TLRs play a key role in microbial recognition and control of adaptive immune responses, which have received widespread attention. Many studies demonstrate that MYD88 is the main linker molecule of the TLRs signal transduction pathway. TLRs combine with MYD88 through the Toll-IL-1 receptor (TIR) domain in the intracellular region to initiate the cascade reaction pathway and then promote the regulation of downstream signaling pathways (57). Most studies suggest that the TLRs/MYD88 signaling pathway plays an important role in restricting the penetration of the intestinal microbiome and preventing mucosal immune regulation disorders, and is essential for maintaining a normal intestinal mucosal barrier and regulating intestinal homeostasis (11).

TLRs are expressed in most intestinal epithelial cells, and the activation of TLRs is related to the development and maturation of secretory intestinal epithelial cells, especially goblet cells. TLRs have multiple family members, and it has been confirmed that TLRs may influence mucosal injury and intestinal inflammation by regulating the integrity of the intestinal mucosal barrier, which provides a target for modulating the intestinal mucosal barrier (58). Under normal circumstances, TLRs can promote the secretion of AMPs and antibacterial factors such as RegIIIγ to exert antibacterial effects, and also promote the uptake and transcellular action of SIgA from the lamina propria to the lumen, thereby promoting the immunomodulatory function of the intestinal mucosal immune barrier (59). In addition, TLRs promote the proliferation of intestinal epithelial cells and induce the epithelial response, which helps to maintain and repair intestinal epithelial cells. It is worth noting that the regulation of tight junctions by TLRs is two-sided (60). Among them, TLR2 plays an active role on regulating the integrity of the tight junctions, while TLR4 plays a negative role. TLR2 recognizes and binds LPS, then promotes the secretion of the mucus layer and the transfer of ZO-1 to the top of cells to strengthen tight junctions through the TLR2 signaling pathway (61, 62). On the contrary, the signaling pathway of TLR4 leads to the contraction of cytoskeletal and relaxes the tight junctions to enhance the permeability of the intestinal mucosal barrier (63, 64).

In a pathological state, the dysregulation of the TLRs signal pathway causes the reduced proliferation of intestinal epithelial cells and the secretion of antibacterial substances (65) and results in the dysfunction of the intestinal mucosal barrier, which in turn destroys the immunity steady-state and increase the susceptibility to autoimmune diseases. Further study has found that the destruction of the TLRs signaling pathway in MYD88 gene-deficient mice leads to the dysfunction of intestinal epithelial cells and aggravates intestinal inflammation. And the targeted deletion of MYD88 in intestinal epithelial cells results in compromised antibacterial immunity associated with the downregulation of mucin-2 and AMPs (66). *In vitro* experiments, the effect of TLRs signaling pathway on intestinal mucosal barrier function was evaluated by Caco2 adenocarcinoma cell line, which is derived from the human colonic adenocarcinoma cells and is used as intestinal epithelial cell model (67). It was found that the knockout of specific components of the TLRs signaling pathway could lead to decreased proliferation of intestinal epithelial cells. Thus, the TLRs/MYD88 signaling pathway is important in regulating the dynamic balance of Intestinal epithelial cells and maintaining the intestinal mucosa barrier.

The above proves that the TLRs signaling pathway plays an important role in maintaining the integrity of the intestinal mucosal barrier and exerting antibacterial function by inducing the proliferation and maturation of intestinal epithelial cells and the secretion of antibacterial substances, which depends on the tight regulation mechanisms. The abnormal TLRs signal pathways induce excessive responses by influencing the normal intestinal mucosal barrier.

**Figure 2:**
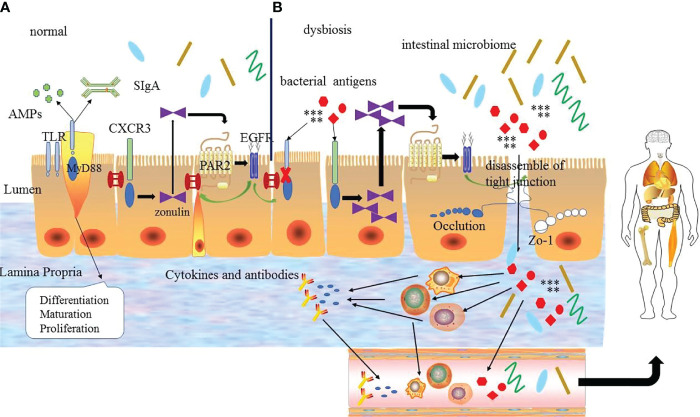
**(A)** The regulation mechanism of the intestinal mucosal barrier. Under normal circumstances the intestinal mucosal barrier depends on the normal zonulin pathway, intestinal microbiome and Toll-like receptors signal pathway. TLRs can regulate the differentiation, proliferation and maturation of intestinal epithelial cells, zonulin reversibly regulates tight junctions *via* zonulin pathway and the intestinal microbiome regulates the normal microecological environment, which interacts with each other to guarantee the normal intestinal mucosal barrier function. **(B)** Under abnormal conditions, bacterial antigens and disordered intestinal microbiome make the TLR signaling pathway abnormal, and the latter destroys the intestinal epithelial cells. At the same time, it can also increase the production of zonulin to promote the zonulin pathway and lead to the destruction of tight junctions ultimately. Bacterial antigens and intestinal microbiome enter into the intestinal lamina propria through the intestinal epithelial cells to interact with immune cells leading to the production of inflammatory factors and abnormal antibodies, which enter the blood circulation and finally enter the body tissues to promote the development of diseases. EGFR: epidermal growth factor receptor; PAR2: protease-activated receptor 2.

In conclusion, the zonulin pathway, intestinal microbiome, and TLRs signaling pathway play an important role in regulating the intestinal mucosal barrier, body homeostasis, and inflammation to some extent. And these regulatory mechanisms interact with each other to form a complex network to adjust and maintain the function of intestinal mucosa barrier, rather than act independently (**Figure 2**). The specific mechanism has not been fully studied and needs further exploration.

## Intestinal Mucosal Barrier Function Is Involved in the Development of Autoimmune Diseases

The intestinal mucosal barrier can effectively prevent intestinal bacteria and their toxins, antigens, and other substances from entering into body, and maintain the stability of the environment in body. Once the intestinal barrier function is impaired, it may trigger an uncontrollable autoimmune-inflammatory response, which has been associated with the pathogenesis of a variety of autoimmune diseases, such as RA, SLE, AS, IBD, and so on.

### RA

RA is a highly disabling autoimmune disease, and its pathogenesis is still unclear. Current studies believe that genetic and environmental factors lead to the occurrence and development of RA, and more and more studies believe that mucosal immunity, as one of the important factors, cannot be ignored in the progression of the disease (68). Some studies have found that abnormal antibodies can be detected in the serum of RA patients before the onset of clinical arthritis, suggesting that the onset may originate from a site far away from the synovium of the joints. And the changes of the intestinal mucosal barrier function and the increase in intestinal permeability may promote immune cells to enter the joint synovium, which is related to the pathogenesis of RA (69, 70). Based on this, the hypothesis of intestinal mucosa origin was proposed.

Studies have found that RA patients have abnormal intestinal mucosal barrier permeability. Collagen-induced arthritis (CIA) mouse model is used to evaluate the changes in the intestinal mucosal barrier, and it can be observed that an obvious imbalance of the intestinal microbiome and mucosal inflammation occurs in the early stage of CIA mice, and the concentration of cytokines such as IL-17A, IL-22 and IL-23 increased (71). The above study supports that the mucosal immune response can promote the development of inflammatory arthritis. The work of Tajik’s team showed the importance of zonulin in the pathogenesis of RA and revealed that zonulin may be a potential therapeutic target for the treatment of RA (50). They found that the level of zonulin and intestinal permeability increased significantly before the clinical onset of arthritis by observing the changes of the function and serum markers of the intestinal mucosal barrier during the modeling of CIA mice. And further research found that after the treatment of CIA mice with butyrate-containing drinking water, the intestinal permeability was improved and the expression level of mouse tight junction protein mRNA was increased while the concentration of serum zonulin was reduced. And after the treatment of CIA mice with the larazotide (a zonulin antagonist), the inflammation of the synovial membrane and the number of osteoclasts was relieved. On the contrary, the use of the zonulin agonist AT-1002 led to the aggravation of symptoms (50), which suggested that the dysfunction of the intestinal mucosal barrier caused by the excessive signal transduction of the zonulin pathway promotes the progression of the disease. The above research results confirm that the dysfunction of the intestinal mucosal barrier is a key event to transform autoimmunity into inflammation, which is involved in the occurrence and development of RA. And the treatment of targeted recovery of the intestinal mucosal barrier is beneficial to disease improvement.

### SLE

SLE is a multi-system autoimmune disease characterized by the secretion of multiple autoantibodies, which leads to the involvement of multiple organs. The etiology of SLE is still unclear. But in recent years, evidences about the destruction of the intestinal mucosal barrier have been found in SLE, indicating that the destruction of the intestinal mucosal barrier leads to chronic inflammation and autoimmune activation.

It has shown that there was an imbalance in the intestinal microbiome in patients with SLE and the translocation of *Enterococcus gallisepticum* was detected in the liver of patients with SLE *in vitro* tissue culture, which indicated that the bacteria could cross the intestinal barrier of lupus mice to enter the liver, spleen and other tissues to promote the autoimmune response (72), which may be an important mechanism for the development of SLE. Several downstream proteins in the TLR signaling pathway are highly relevant to the pathogenesis of SLE and are potential therapeutic targets, including MYD88, Interleukin-1 Receptor-Associated Kinases (IRAKs), and IFN-α. LPS, mostly coming from the intestinal lumen, is recognized by TLR4 and the interaction between them has been proven to promote the inflammatory response and exacerbate the development of SLE (73). And studies found that the high level of plasma LPS in patients with SLE caused by the impaired intestinal mucosal barrier was positively correlated with the level of serum anti-double-stranded DNA (anti-dsDNA) antibodies, which suggested that the increased intestinal permeability was beneficial to LPS to penetrate the intestinal epithelium and translocate into the tissue to promote the progression of disease (74, 75). In lupus mouse models by inducing gut leakage have observed the production of anti-dsDNA antibodies and the deposition of circulating immune complex (CIC) to aggravate the disease, which suggests that the increase in intestinal permeability caused by dysfunction of the intestinal mucosal barrier promotes the progression of lupus (76).Therefore, the dysfunction of the intestinal mucosal barrier is one of the important factors in the development of SLE. Maintaining the integrity of the intestinal mucosal barrier function is of great significance for preventing the microbiome from crossing the intestinal wall into the circulation or other tissues and improving the disease. But the exact role has not yet been determined, which still needs in-depth investigation.

### AS

AS is a common chronic inflammatory disease characterized by a stiff and painful back, which always affects sacroiliac joints and the spine with or without systemic symptoms (77). The etiology of AS remains unclear and environmental factors, especially the intestinal microbiome plays an essential role in the pathogenesis of AS. It has been confirmed that the diversity and abundance of the intestinal microbiome of patients with AS have great changes, and may trigger autoimmunity through molecular mimicry mechanisms (78). And the connection between the intestinal mucosal barrier and the syndrome has been established. The study of patients with AS has found that the level of zonulin in the serum of patients with AS was elevated, and the up-regulated zonulin changed the expression of tight junctions between intestinal epithelial cells to lead to the systemic symptoms (49), which may be the therapeutic target of AS.

### IBD

IBD is a chronic non-specific inflammatory disease, including Crohn’s disease (CD) and ulcerative colitis (UC). In recent years, a large amount of evidence suggested that the dysfunction of the intestinal mucosal barrier played an important role in the pathogenesis of IBD (79–81). It has been found that the increase in the permeability of the intestinal mucosal barrier in Crohn’s disease occurs before clinical recurrence. The first-degree relatives of patients with Crohn’s disease have found increased intestinal permeability, and most of these healthy first-degree relatives will develop into IBD eventually (80), which indicates that intestinal mucosal barrier dysfunction is the first event of the disease. And the level of serum zonulin was higher in patients with IBD patients compared to healthy controls (81), which is highly sensitive for the evaluation of intestinal permeability of IBD. The dysfunction of the intestinal mucosal barrier can cause the direct interactions between the untreated antigens and the mucosal immune system leading to abnormal immune responses, which promotes the further development of the disease. Therefore, the dysfunction of the intestinal mucosal barrier plays a central role in the pathogenesis of IBD and is an important factor leading to the development of IBD, which provides a direction for exploring new strategies for prevention and treatment.

### T1D

T1D is an autoimmune disease characterized by the destruction of insulin-producing beta cells in the pancreas, which is mediated by auto-reactive T cells. Despite there are considerable progresses in the pathogenesis of T1D, the mechanisms of the activation of T cells to mediate the autoimmune response of insulin β cell remains unclear (82). Many studies have found that patients with T1D often presented intestinal inflammation, even in the preclinical phase (83, 84). It seems that intestinal inflammation participates in the progression of the disease. The study of Rouland et al. (84) supported that intestinal inflammation rather than hyperglycemia was the important driver for the development of T1D. They found that the production of IL-17A, IL-22, and IL-23A by intestinal immune cells was decreased during the progression of T1D in non-obese diabetic (NOD) mice. This result was associated with the intestinal inflammation and the dysbiosis of intestinal microbiome such as segmented filamentous bacteria (SFB), and the application of anti-inflammation treatment could prevent the development. While the hyperglycemic mice without inflammation did not promote the progression of disease, because it did not lead to the alteration of the intestinal mucosal barrier and the dysbiosis of intestinal microbiome, which suggested that inflammation was the important driver in the development of T1D (84). The intestinal inflammation of T1D is mainly the dysfunction of intestinal epithelial cell and the loss of gut integrity, namely the impaired intestinal mucosal barrier.

With the discovery of the intestinal mucosal barrier in T1D patients, there are abundant evidences supporting that the impaired intestinal mucosal barrier mediates the autoimmune response of insulin β cells and is involved in the occurrence and development of T1D (85, 86). Consistent with this, the study of Sorini et al. (87) found that diabetic NOD mice exhibited increased intestinal permeability and the loss of intestinal mucosal barrier integrity to promote the activation of islet-reactive T cells in the gut mucosa, which migrate to pancreatic tissues and induce autoimmune diabetes. And their findings demonstrated that the impaired intestinal mucosal barrier could be a trigger to promote the activation of islet reactive T cells and induce autoimmune diabetes. In addition, the altered composition of the intestinal microbiome is also involved in the onset of T1D (88–90), which is related to the impaired intestinal mucosal barrier or accelerates the disruption of the intestinal mucosal barrier directly. Zonulin as a regulator of tight junction in the intestinal mucosal barrier has been reported to participate in the pathogenesis of T1D (91–93). The increased serum concentration of zonulin was found in the diabetic rat model, and the elevated zonulin levels were detected in the intestinal tissue of T1D patients, which was positively correlated with the disease progression (91). Besides that, the study found that patients with T1D and their relatives had the high level zonulin (92), which indicated that the high zonulin increased the susceptibility to T1D and its pathways have a pathogenic effect on T1D. And a study on animal models prone to T1D (93) showed that the impaired intestinal mucosal barrier occurred before the onset of the disease, and the addition of zonulin antagonists reversed the intestinal mucosal barrier disorder and improved the T1D performance of disease-prone rats.

The above propose that the intestinal mucosal barrier plays a vital role in body and the impaired intestinal mucosal barrier contributes to be a factor to promote the progression of T1D. Maintaining the intestinal mucosal barrier function can be used as a preventive strategy for T1D.

### Multiple Sclerosis

T cell-dependent demyelination of neurons is the pathological basis of multiple sclerosis (MS), which is affected by multi-factors including genetic and environmental influences. Recently, the link between the blood brain barrier and the intestinal mucosal barrier has been investigated. Especially the influence of the intestinal mucosal barrier on the pathophysiology of MS has been described (94–97). Studies have found a translocation of bacteria or bacterial products from the intestines into the circulation were present in MS in the absence of gastrointestinal diseases (94, 95), which implicated that the disruption of the intestinal mucosal barrier was involved in the pathogenesis of MS. And the increased level of zonulin observed in MS rapidly increased the permeability of the blood-brain barrier and intestinal mucosal barrier by destroying tight junctions (96), which further indicated that the breakdown of the intestinal mucosal barrier participates in the MS. A study about the microbiome on the effect of experimental autoimmune encephalomyelitis (EAE)(an animal model of MS) showed that germ-free mice had the protective effects in either delayed onset of symptoms or complete protection from disease (94), suggesting the composition of the microbiome was associated with the progression of MS. The brain-gut axis abnormalities provide a new avenue for therapeutic targets for the treatment of MS.

## Conclusion and Outlook

The intestinal tract is an important digestive and absorption organ of the human body. As a semi-osmotic barrier, it can not only provide water and nutrients that are necessary for the survival of organisms through material uptake, but also can resist the passage of various potentially harmful substances to maintain the balance of the internal environment of the body. The regulation of this seemingly conflicting task is achieved by the function of the intestinal mucosal barrier. As an important defense system of the body, the intestinal mucosal barrier can prevent pathogens from entering the body to maintain the immune balance. When the function of the intestinal mucosal barrier is impaired, it can stimulate abnormal immune responses and promote the occurrence and development of autoimmune diseases, which can be used as a predictive marker of the pathophysiology of autoimmune disease. Considering the contributions of the impaired intestinal mucosal barrier to inflammation and multiple autoimmune diseases, reversing the impaired intestinal mucosal barrier appears to be an attractive therapeutic strategy. Targeting the intestinal mucosal barrier is promising for the treatment and prevention of diseases. Therefore, more and more attention has been paid to finding and developing new therapeutic targets to improve intestinal mucosal barrier function. However, therapeutic intervention in the regulation of intestinal mucosal barrier function is still an emerging topic, and more studies are needed to understand the role of intestinal mucosal barrier function in various diseases. Maybe the zonulin pathway, the intestinal microbiome, and TLRs signal pathway are important checkpoints for intestinal mucosal barrier intervention. It is necessary for us to conduct in-depth studies to provide good intervention targets for the clinical treatment of autoimmune diseases to benefit patients.

## Author Contributions

AJ drafted the manuscript, prepared illustrations, and discussed the content with the other authors. CJ conceived the topic and revised the content of the manuscript. LY and WY intellectually revised the manuscript and searched the literature. FR, HX, ZF and YJ also revised the content of the manuscript. All of the authors approved the manuscript for publication.

## Funding

This study was supported by Key Research and Development (R&D) Projects of Shanxi Province (Social Development) (201903D321143).

## Author Disclaimer

This article is an informal communication that represents the authors’ judgment.

## Conflict of Interest

The authors declared that the research was conducted in the absence of any commercial or financial relationships that could be construed as a the potential conflict of interest.

## Publisher’s Note

All claims expressed in this article are solely those of the authors and do not necessarily represent those of their affiliated organizations, or those of the publisher, the editors and the reviewers. Any product that may be evaluated in this article, or claim that may be made by its manufacturer, is not guaranteed or endorsed by the publisher.
